# Validation of a Short Food Frequency Questionnaire to Measure Dietary Intake of a Selection of Micronutrients in Oncology Patients Undergoing Systemic Therapy

**DOI:** 10.3390/nu13124557

**Published:** 2021-12-20

**Authors:** Mitali S. Mukherjee, Shawgi Sukumaran, Christopher L. Delaney, Michelle D. Miller

**Affiliations:** 1 Caring Futures Institute, College of Nursing and Health Sciences, Flinders University, Bedford Park, SA 5042, Australia; michelle.miller@flinders.edu.au; 2 College of Medicine and Public Health, Flinders University, Bedford Park, SA 5042, Australia; shawgi.sukumaran@flinders.edu.au (S.S.); chris.delaney@sa.gov.au (C.L.D.); 3 Department of Medical Oncology, Flinders Medical Centre, Bedford Park, SA 5042, Australia; 4 Department of Vascular Surgery, Flinders Medical Centre, Bedford Park, SA 5042, Australia

**Keywords:** anti-inflammatory, micronutrients, FFQ, oncology, chemotherapy, immunotherapy, cancer

## Abstract

Dietary intake, specifically consumption of anti-inflammatory micronutrients, can play a role in both cancer initiation as well as the treatment-related outcomes experienced by patients receiving systemic cancer therapy. Increasing research is being conducted to determine whether micronutrient supplementation can aid in altering the tumor microenvironment (TME), reducing inflammatory side effects and immune-related adverse events (irAEs). However, further research pertaining to the adequacy of dietary micronutrient intake is indicated in the oncology cohort. Currently, no tool measuring dietary intakes of various micronutrients exists in the oncology population. In this study, a 21-item food frequency questionnaire (FFQ) measuring intakes of 14 different micronutrients was validated using diet history as the reference method in 112 oncology patients. Bland Altman plot and Passing Bablok regression analysis were conducted to determine agreement between the two methods. The results showed adequate agreement between FFQ and diet history for 12 nutrients including copper, iron, vitamins A, E, and D, alpha linolenic acid (ALA), long-chain omega 3 fatty acids (LC n3-FA), arginine, glutamic acid, isoleucine, leucine, and valine. This 21-item FFQ, which takes an average of 10 min to complete, can be utilized as a quick screening tool to determine adequacy for 12 different micronutrients in place of a diet history.

## 1. Introduction

Cancer is the leading cause of death globally, accounting for 10 million deaths and 19.3 million new cases in 2019 [1]. It is established that chronic inflammation is a risk factor for cancer [2]. Cancer initiation may occur due to sustained inflammation inducing genetic instability through cytokine signaling or activation of reactive oxygen species (ROS) [3]. The mechanism of cancer-related inflammation involves intrinsic and extrinsic pathways. In the intrinsic pathway, oncogenes drive the transformed cells to produce cytokines, chemokines, and growth factors. They also induce the expression of cyclooxygenase-2 (COX-2), which allows tumor cells to produce prostaglandins. These factors contribute to tumor proliferation. They can contribute to generation of a tumor microenvironment (TME) through recruitment of inflammatory cells. The extrinsic pathway is mediated by the inflammatory cells and hypoxia in the TME. Disruptions in the normal tissues lead to the secretion of damage-associated molecular patterns (DAMPs). The recognition of these DAMPs by toll-like receptors leads to the secretion of pro-inflammatory cytokines such as tumor necrosis factor (TNF), interleukin-1 (IL-1), and interleukin-6 (IL-6), which furthermore activate nuclear factor kappa B (NF-kB) and signal transducer and activator of transcription 3 (STAT3), which in turn promote tumor proliferation. A hypoxic environment also promotes expression of oncogenes and can act as a source of DAMPs. Overall, this amplifies cancer-related inflammation [4]. Furthermore, data from animal models and human studies have shown that cytokines can have an impact on symptoms such as anorexia or cachexia, pain, nausea, weakness, and sedation experienced by patients with advanced cancer [5]. A recent study involving 90 cancer patients showed that overall survival was shorter in immunotherapy patients who were sarcopenic and with elevated inflammation [6,7].

Diet has been recognized as a factor affecting inflammation [8]. Dietary factors are associated with risk of developing cancers, especially breast, colorectal, head or neck, lung, and prostate [9]. A dietary pattern consisting of high quantities of fruits and vegetables, lean meats as opposed to red and/or processed meats, fish, wholegrains as opposed to refined or processed foods, and healthy fats is globally accepted as favorable [10]. A prospective cohort study including 100,881 participants followed participants until diagnosis of an invasive cancer. The results showed that participants that had a lower dietary inflammatory index (DII) score and a greater adherence to the Mediterranean diet, which is rich in antioxidants and anti-inflammatory foods, had an overall lower risk of cancer incidence [11].

Apart from cancer initiation, dietary factors also play a role in side effects and treatment outcomes experienced due to cancer therapy. Formation of ROS is a primary mechanism of chemotherapy drugs used to destroy cancer cells. The production of these free radicals can lead to serious side effects [12]. The interactions between chemotherapy drugs and antioxidants are a complex area, and factors such as dose, localization, and metabolism of drugs may affect the development of ROS. Use of micronutrients and dietary supplements during chemotherapy and radiotherapy has been a cause for concern from an oncological point of view as antioxidants, for instance, Vitamin C, are thought to reduce the effectiveness of chemotherapy and radiotherapy by protecting cells against damage [13]. However, recent studies have shown increasing evidence that the provision of selected micronutrients could lead to a better response to cancer therapy due to fewer side effects experienced and lower treatment discontinuation rates [12]. Research using supplementation of vitamins such as A (or retinol equivalents), B, C, and D (or cholecalciferol) and minerals such as iron, zinc, and selenium suggest that they may aid in reducing immune-related adverse events (irAEs) in patients receiving immunotherapy by enhancing immune response rates and reducing oxidative stress [14,15]. Moreover, it has been estimated that 30% to 90% patients diagnosed with cancer often consumed micronutrient supplements without the knowledge of their oncologists [16]. Existing literature has suggested that vitamins A, D, C, and E, long-chain omega 3 fatty acids (LC n3-FA), alpha linolenic acids (ALA), and zinc may have an anti-inflammatory role [17,18,19,20,21]. Iron, on the other hand may exert an inflammatory role [21]. Conflicting information exists relating to copper and branched chain amino acids (BCAA) [22,23,24,25,26].

A question, therefore, arises as to whether micronutrient intakes in diet with potential anti-inflammatory and antioxidant benefits can have an impact on disease and side effect outcomes.

To our knowledge, no dietary tool exists in the oncology population that collectively and quickly assesses fourteen micronutrients including copper, iron, zinc, retinol equivalents, vitamin C, vitamin D, vitamin E, ALA, LC n3-FA, arginine, glutamic acid, isoleucine leucine, and valine [27,28,29,30,31].

Development and validation of such a tool in the oncology population will pave a pathway for further research into dietary intake of micronutrients and their consequent effects on disease outcomes. In addition, studying single nutrients may have limitations such as small effect size and insufficient statistical power. A tool assessing multiple nutrients will account for the synergistic effects of multiple micronutrients [8].

The aim of this study was to validate an FFQ that measures various micronutrients against an accepted reference method. It is hypothesized that adequate agreement will be demonstrated between the two methods that is clinically meaningful, so accurate and quick measurement of micronutrient intake is possible.

## 2. Materials and Methods

Ethics approval was obtained for this study on 22 October 2020 from Southern Adelaide Clinical Human Research Ethics Committee (SAC HREC).

### 2.1. Study Population and Data Collected

A sample of 112 patients receiving chemotherapy, immunotherapy, or combined systematic cancer therapies were approached for participation via convenience sampling method at Flinders Infusion Suite, Flinders Medical Centre, Adelaide, South Australia. Recruitment occurred from 24 October 2020 until 27 August 2021. Patients above the age of 18 years, diagnosed with cancer, and on an oral diet were invited to participate in the study, whereas those with recall deficits or barriers to complete an interview-administered FFQ and diet history were excluded from participation. Data pertaining to age, gender, type of systemic treatment received, co-morbidities, infusion cycle regimen, and time required to complete the FFQ were collected.

### 2.2. Administration of FFQ and Diet History

On receiving written consent for participation in the study from participants, an accredited practicing dietitian (APD) and PhD candidate (MSM) administered both the FFQ and diet history. The FFQ was interview-administered in an open-ended manner with descriptions of reference food serve sizes first. The duration to complete the FFQ was determined using a stopwatch. This was followed by the compilation of a diet history. Detailed information pertaining to frequency, quantity, and types of all foods and drinks consumed in a typical week, most representative of the patient’s usual diet, was collected. Administering the test method (FFQ) first ensured that it would be encountered as an independent dietary assessment and that the reference method (diet history) would not draw the participants’ attention to their diets beforehand [17].

### 2.3. Development of Food Frequency Questionnaire

The original version of this semi-quantitative food frequency questionnaire was a 20-item questionnaire aiming to assess intake of 14 different micronutrients. It was developed and piloted in the peripheral arterial disease (PAD) population at Flinders Medical Centre in 2019 using a sample of 102 participants. The results from the first validation study in the PAD population showed that the original version overestimated vitamin C, vitamin E, and amino acids while underestimating iron, zinc, and retinol equivalents. Modifications were made to the original version to develop an updated version in 2020. These modifications included addition of root vegetables that are rich in retinol equivalents, separation of fruits based on their vitamin C content, and inclusion of sub-questions where relevant to determine the type of food consumed. The updated version demonstrated clinically meaningful agreement between the FFQ and diet history methods for all nutrients except zinc [32].

The FFQ used in the current study in the oncology population is the same as the 2020 updated version validated in the PAD population (Appendix A). It is a 21-item questionnaire aimed at assessing average daily intakes of 14 different micronutrients, namely copper, iron, zinc, retinol equivalents, vitamin C, vitamin D, vitamin E, ALA, LC n3-FA, arginine, glutamic acid, isoleucine, leucine, and valine over the past 12 months.

The updated FFQ was developed in the following steps. Similarly to the PAD population, the age at which incidence of cancer increases is 51 and over [33]. Based on this information, firstly, commonly consumed food items in the Australian population of the 51-and-over age group were determined using the Australian Health Survey- Nutrition first results (AHS) [34]. Secondly, in conjunction with information from AHS, food items with high levels of micronutrients were determined using previous literature and the Australian food composition database release 1 [35]. Finally, using data obtained from the 2019 pilot study in the PAD population, further modifications, such as grouping foods consisting of similar micronutrient compositions and adding sub-questions where relevant, were completed to create a 21-item semi-quantitative questionnaire [32].

The data collected from the semi-quantitative questionnaire pertained to frequency as well as portion sizes consumed. For every question, a reference portion size description was provided in either grams, cups, teaspoons, tablespoons, or slices. Foodworks software was utilized to determine the accuracy of these portion sizes. The first five questions collected information on the average portion of bread, milk, margarine, oil, and fruit consumed per day over the past 12 months. Sub-questions were included to determine the type of food consumed where relevant, for instance, wholegrain, white, or whole meal bread. The next sixteen questions asked about the frequency of consumption of food items such as juice, breakfast cereals, other cereals, potato, sweet potato or carrot, tomato products, leafy green vegetables, peas, cruciferous vegetables, red meat, lean meat, oil fish, white fish, eggs, cheese, and nuts over the past 12 months. Furthermore, for all 16 of these food items, portion sizes consumed were also determined. Sub-questions to determine the type of food consumed were also asked where relevant (refer to Appendix A).

### 2.4. Rationale for Use of Diet History as Reference Method

Diet history was deemed the most appropriate reference method for validating the FFQ in the oncology population. A diet history, collected through an open-ended interview, provides detailed information about the usual dietary consumption in a single interview. It collects information on a person’s food and fluid intake from the first to last meal or drink, in a day, over a period of time—a typical week in our study [36]. Diet history can provide a good overview of dietary intake over a long period of time, usually up to a year [37]. In contrast, multiple 24-h recalls, weighed food records, long food frequency questionnaires, and food diaries can be onerous and time-consuming for participants to complete [37]. Impact on foods and quantities selected due to burden is a disadvantage for weighed food records and food diaries. Recall bias is a possible disadvantage of 24-h recalls and food frequency questionnaires [37]. Moreover, a single 24-h recall may not be representative of an individual’s usual diet. Biomarkers can be expensive to measure and may also be affected by factors apart from food intake [38]. As there was no food frequency questionnaire measuring the 14 nutrients measured in this FFQ, using another FFQ as a reference method was not feasible.

### 2.5. Databases and Data Entry Process

Two databases were created for the documentation of dietary information from the FFQ and diet history. Both databases were designed to display values of the 14 micronutrients consumed as amounts consumed per day.

The Australian Food Composition Database release 1 was utilized to convert nutrient values per 100 g to nutrient values per serve identical to the described reference serve sizes on the FFQ [35]. Using two columns, namely frequency of consumption and serves consumed, the calculation of nutrients consumed per day was completed.

The diet history database used was a modified version of the Australian Food Composition Database release 1 that provided nutrient values per 100 g for 1534 food items. The average weight in grams of food items consumed per day was first determined using the Foodworks software through logging in participants’ diet histories. This weight of individual food items consumed on average per day was then inserted into the database to calculate the nutrient values consumed per day.

### 2.6. Statistical Analysis and Clinical Significance

IBM SPSS Statistics Version 25 was used to express descriptive statistics such as age, gender, treatment type, and time taken to complete questionnaire as frequency or mean and standard deviation and to determine whether results were normally distributed. To determine agreement between the FFQ and diet history, STATA IC version 15.1 was used to generate Bland–Altman plots. The data were normally distributed. For the limits of agreement (LOA) to be estimated precisely, the Bland–Altman method requires a minimum sample size of 50 and preferably a sample size of 100 or more. Therefore, a sample size of 112 was adequate for this analysis [17]. The difference in intake of each nutrient (FFQ–diet history) on Y-axis was plotted against the mean intake of each nutrient (FFQ+diet history/2) on X-axis. The mean difference (bias), upper and lower limits of agreement, and their respective 95% confidence intervals were determined and plotted [39]. Passing–Bablok regression was completed to determine proportional bias [40]. If the 95% confidence intervals (CI) for intercept included value 0, it could be inferred that there was no constant difference between the values obtained from the two methods. If the 95% CI for the slope included value 1, it could be inferred that there was no proportional difference between the two methods [41]. A cusum test P value above 0.05 indicated no significant difference from linearity and that the data points were randomly distributed below and above the regression line [41].

It was predetermined that for the oncology population, the results would be deemed clinically significant if the difference between the micronutrient intakes calculated using the FFQ and diet history was less than a serve of a food containing high levels of the respective micronutrients. The LOA were deemed clinically meaningful at less than or equal to 2 serves of food containing high levels of the respective nutrients.

## 3. Results

### 3.1. Participant Characteristics

The short FFQ and diet history were interview-administered in person to 112 participants at the Flinders Infusion Centre. Patients consuming an oral diet with solid cancers such as liver, prostate, lung, bladder, bowel, skin, colorectal, head and neck, esophageal, breast, pancreatic, brain, kidney, thyroid, endothelial, and ovarian cancer or leukemia were included. The age of participants ranged from 21 to 88 years. The time taken to complete the FFQ ranged from 5 to 18 min. Participant characteristics are summarized in Table 1.

### 3.2. Agreement between FFQ and Diet History

The biases for all nutrients barring vitamin C were within the clinically acceptable ranges and also within 95% CI as shown in Table 2. A negative bias was observed for three nutrients, namely iron, zinc, and LC *n*-3 FA, whereas all other nutrients demonstrated a positive bias.

As shown in Table 2, the lower LOA were within clinically acceptable ranges and within 95% CI for all nutrients except iron, zinc, vitamin C, and ALA. The upper LOA were within clinically acceptable ranges and within 95% CI for all nutrients except iron, zinc, vitamin C, vitamin E, and ALA.

**Figure 1:**
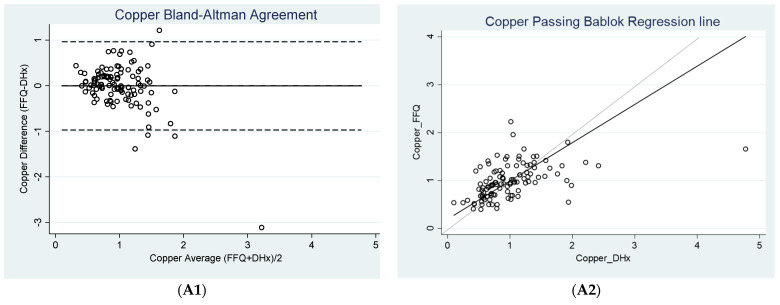

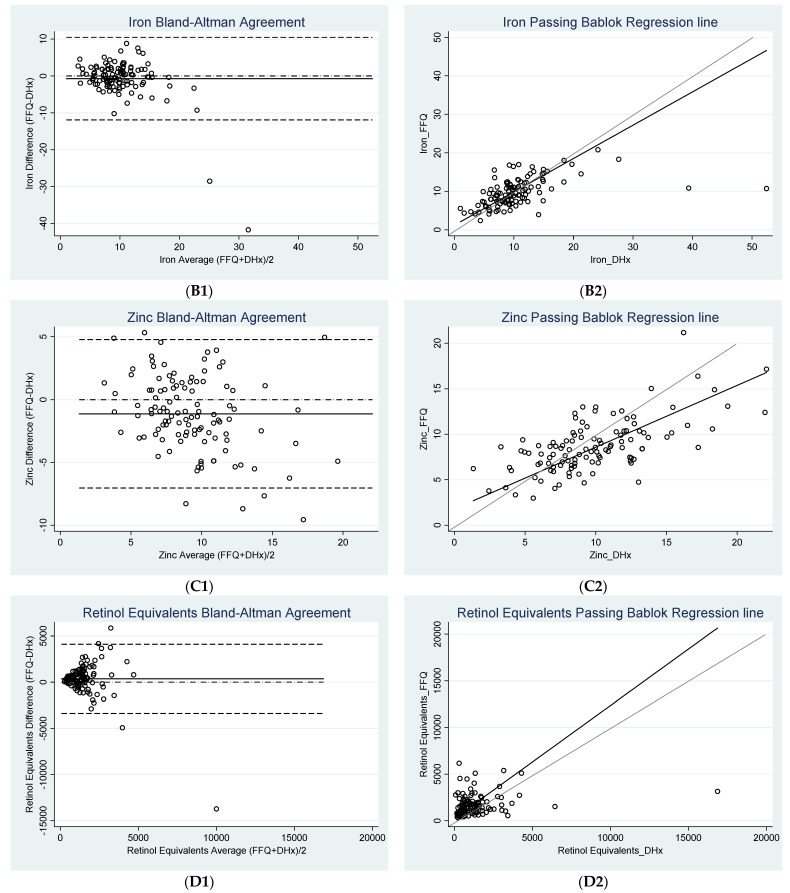

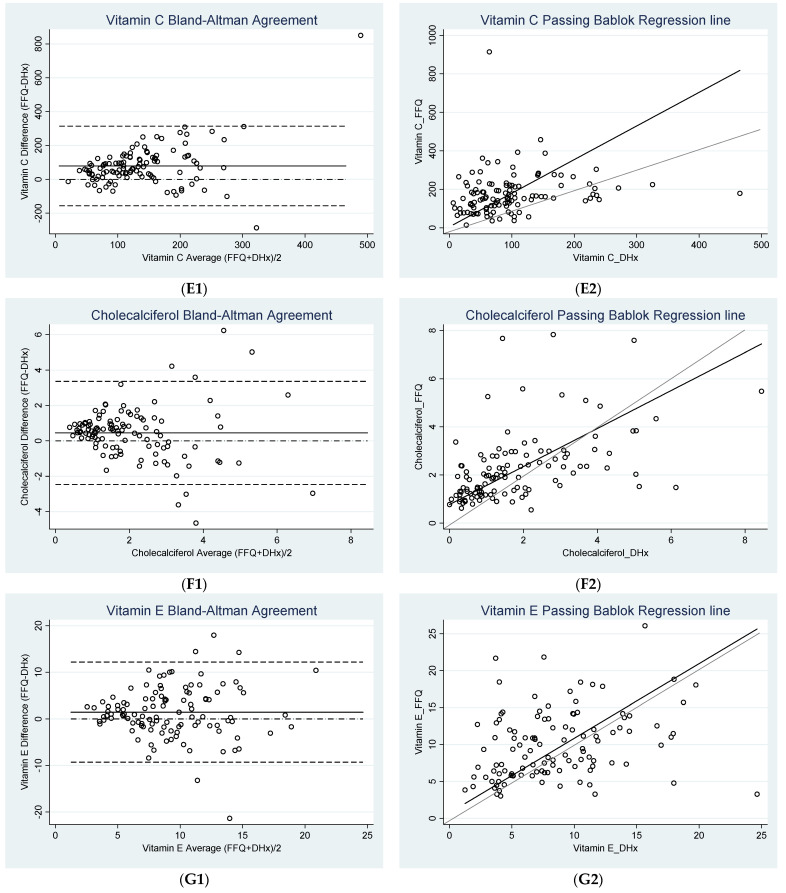

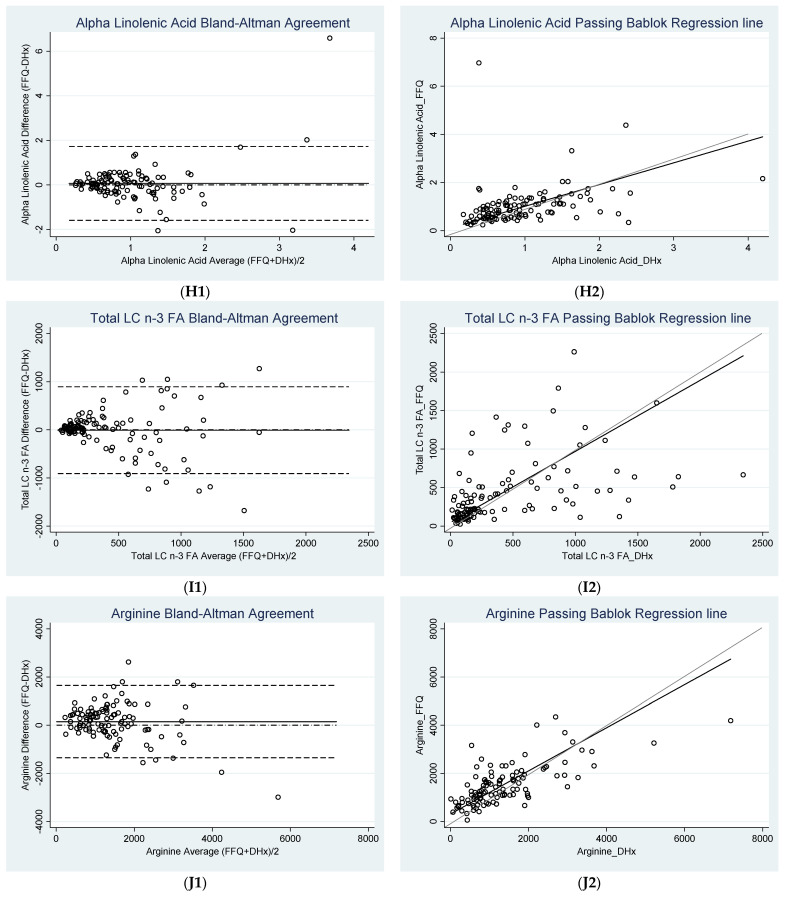

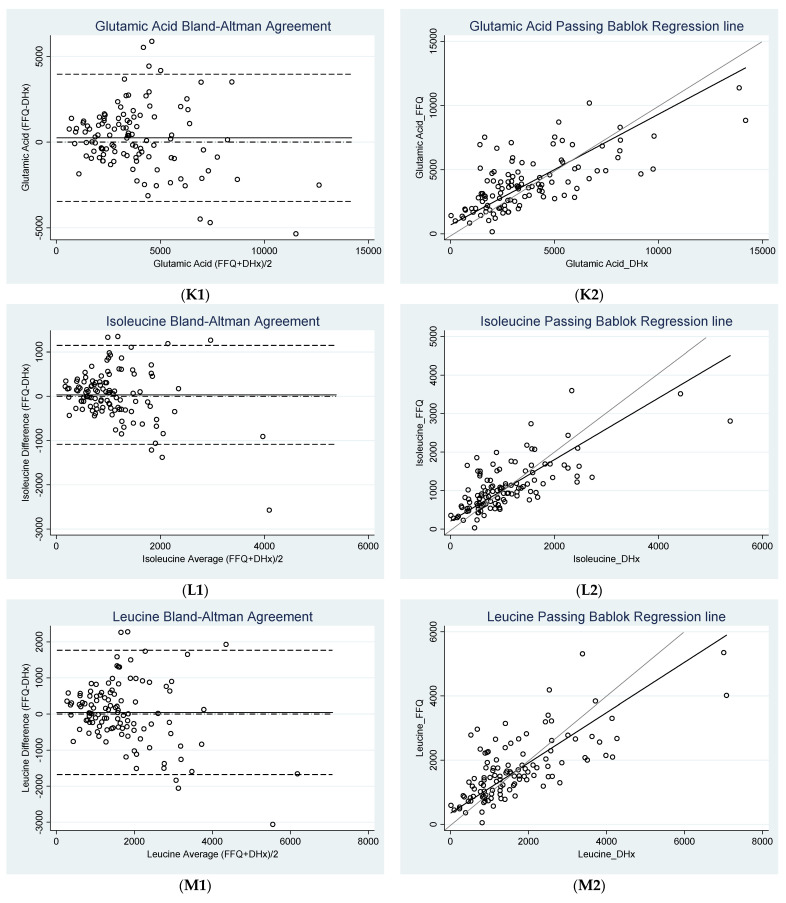

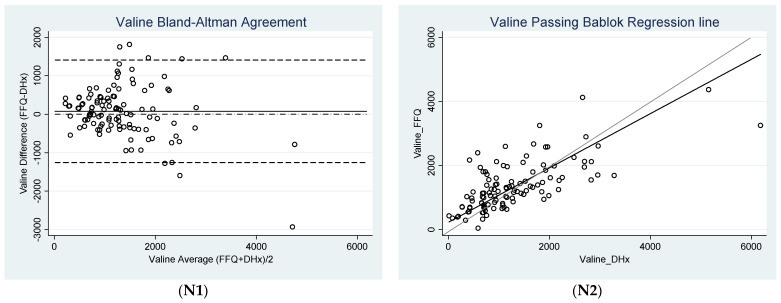
1: Bland-Altman plots showing agreement between the reference method diet history (DHx) and Food Frequency Questionnaire (FFQ) 2: Passing Bablok analysis showing constant difference and proportional difference (black line) against line showing 100% agreement between the two methods (grey line) for (**A**) copper, (**B**) iron, (**C**) zinc, (**D**) retinol equivalents, (**E**) vitamin C, (**F**) cholecalciferol, (**G**) vitamin E, (**H**) alpha linolenic acid (ALA), (**I**) total long chain omega-3 fatty acids (LC *n*-3 FA), (**J**) arginine, (**K**) glutamine, (**L**) isoleucine, (**M**) leucine, (**N**) valine.

No constant difference was observed between the FFQ and diet history for six nutrients including iron, retinol equivalents, vitamin C, vitamin E, ALA, and LC n-3 FA, whereas the remaining eight nutrients demonstrated a constant difference as shown in Table 3 and Figure 1.

No proportional difference was observed between FFQ and diet history for nine nutrients including iron, retinol equivalents, cholecalciferol (vitamin D), vitamin E, ALA, LC n-3 FA, arginine, glutamic acid, and valine, whereas a proportional difference was observed in the remaining nutrients as shown in Table 3 and Figure 1.

There was no significant difference from linearity between the two tools for all nutrients except vitamin C, isoleucine, leucine, and valine as shown in Table 3.

## 4. Discussion

This study carried out the validation of a 21-item short FFQ assessing intake of 14 different micronutrients against the reference method, diet history. Micronutrient intake can have an impact on inflammation and TME and can consequently also influence inflammatory side effects and irAEs [2,8,9,11]. In the oncology population, literature shows that micronutrient intake is related to treatment-related outcomes [12,13]. Increasing research is being conducted in this area to determine the efficacy of micronutrient supplementation on managing side effects and improving treatment effectiveness [14,15,16]. Therefore, this research is important to derive information about the adequacy of dietary intake of micronutrients in this population group.

Except for vitamin C, all other micronutrients, namely copper, iron, vitamins A, E and D, zinc, ALA, total LC n-3 FA, arginine, glutamic acid, isoleucine, leucine, and valine, showed a bias well within the pre-defined clinically acceptable bias range. Furthermore, the biases for all of these nutrients remained within the 95% confidence intervals. Overall, the results suggested adequate agreement for all nutrients except vitamin C.

However, proportional bias was present for five nutrients measured including copper, zinc, vitamin C, isoleucine, and leucine. As seen from Figure 1, a large proportional bias was observed for zinc and vitamin C. As compared to the diet history, the current FFQ tended to underestimate zinc intake at higher dietary zinc intakes. These results are similar to the validation study of the same FFQ conducted in the PAD population [32]. In relation to vitamin C, the FFQ overestimated vitamin C intakes, with the overestimate becoming greater as dietary vitamin C intake increased. The proportional bias was slight for copper, isoleucine, and leucine, with a tendency to overestimate respective intakes at lower dietary intakes and underestimate respective intakes at higher dietary intakes. This indicated that intakes determined for zinc and vitamin C should be evaluated with caution.

A recent study validated a zinc-FFQ using a list of foods specifically high in zinc [42]. Foods such as veal, pork and beef liver, pumpkin, and flaxseeds, which are high in zinc, were not specifically included in the current FFQ. In addition, while veal liver contains 8.4 mg of zinc per 100 g, poultry contains only 1.68 mg of zinc per 100 g [42]. As lean meats are grouped together with an average value for each nutrient calculated, the variability of zinc intake depending on the type of meat consumed cannot be accounted for using the current FFQ. Zinc is a key nutrient involved in response to oxidative stress, DNA repair, cell cycle progression, and apoptosis, and zinc levels are dramatically decreased in cancer patients [43]. While limited research is available on the therapeutic benefits of zinc supplementation in cancer therapy, zinc is an important nutrient to consider when evaluating the micronutrient intake adequacy. As this FFQ underestimates zinc, it is less cause for concern as it is likely that the actual dietary intake of patients is greater than calculated. However, further improvements in the FFQ potentially could be made in the future by adding additional questions that segregate foods within grouped foods—for example, by modifying questions relating to cereals or meat intake.

Vitamin C is a micronutrient that is said to be deficient in cancer patients as a result of lower intakes, elevated oxidative stress, and reduced absorbability [44]. Albeit with limited data, supplementing vitamin C orally or via the intravenous route is increasingly being considered for supportive care to prevent cancer-associated symptoms [44]. This makes vitamin C an important nutrient to consider. In context of the vitamin C results, orange and mandarin seasons in Australia span from May to November. As most of the data collection was carried out between October and April, it is likely that the consumption of foods such as oranges and mandarins, which are high in vitamin C, were lower when the diet histories were obtained. Therefore, it is likely that the diet history did not capture the seasonal variation captured by the FFQ. Furthermore, as vitamin C levels are impacted due to several factors in the body, modifications to the FFQ may not be necessary for the oncology population. Instead, should an overall deficit in micronutrients be seen in the FFQ, further information about vitamin C intake could be collected during nutritional assessment. Blood test results will also be pertinent to determine vitamin C adequacy in this population group as other factors affect their levels.

Major strengths and limitations of the FFQ have previously been outlined in a similar study conducted in the PAD population [32]. An additional strength of this study is the utilization of Passing–Bablok analysis in place of linear regression. Passing–Bablok can detect constant difference and proportional difference [41]. At the same time, it is robust against outliers and does not assume that the measurement error is normally distributed [45]. Moreover, the sample size used in this study (*n* = 112) is more than adequate for the minimum sample size (*n* = 40) required to conduct Passing–Bablok analysis [41]. A limitation recognized is that the current FFQ does not collect information about commercial oral nutritional supplements consumed by patients, e.g., Sustagen and Fortisip or protein supplements. These supplements could have additional quantities of micronutrients added to them that are not reflected in the FFQ database. As reflected from the diet histories of the current cohort of this study, only five patients consumed Sustagen and only two patients fortified their foods with protein powder. However, the FFQ could potentially underestimate nutrient intake values when utilized in patients using supplements. Although a question relating to micronutrient supplement intake was asked as part of the FFQ, patients were not able to report the type and dose of supplements taken, thereby limiting any analysis of data relating to supplements consumed. The diet histories were collected at different phases of treatment for different patients. To best manage this limitation, all the patients were asked to provide diet histories representative of their usual diet. However, it is worth considering the possibility of treatment side effects having an impact on the absolute accuracy of the diet history provided. Additionally, this study only included ambulatory patients able to consume 100% of their food intake orally; hence the results of this study may not be considered valid in an inpatient setting. The validation method used in this study was subjective, relying on patients’ memory as opposed to objective reference methods such as blood test results. Although obtaining blood test results is expensive and invasive and may not always provide accurate correlations between dietary intake and biomarker levels, validating the tool against objective measures in the future could further strengthen the findings of this study [17].

The FFQ could pave a pathway for important research that could be meaningful in clinical practice. An immediate use of this FFQ is to quickly screen whether oncology patients are consuming optimal amounts of micronutrients from their diet by comparing their intakes to nutrient reference values (NRVs). Should a deficit or excess be recognized, the screened patient could be offered a dietetic assessment to optimize their nutritional intake. The FFQ could be used as a potential measuring tool in observational research aimed at determining the relationship between dietary intake of nutrients and treatment-related outcomes or side effects. Similarly, it could be used as a measuring tool in observational studies comparing relationships between patients’ micronutrient intake via diet and/or supplementation and treatment-related outcomes. Despite, the heterogeneity in the types of cancers in the current patient cohort, the validation of the FFQ suggests that it has the potential to be validated in various other population groups and may serve as a screener for suboptimal intake of micronutrients in different chronic conditions.

## 5. Conclusions

Given the results, the current FFQ can be considered as a validated screening tool to adequately determine micronutrient intakes of twelve nutrients including copper, iron, vitamins A, E and D, ALA, total LC n-3 FA, arginine, glutamic acid, isoleucine, leucine, and valine in place of a diet history. To determine the adequacy of zinc and vitamin C intake in this setting, further studies may be required.

## Figures and Tables

**Table 1:** Characteristics of oncology participants included in the study exploring the validity of short FFQ against the reference standard diet history.

Characteristics	Mean ± SD or *n* (%)
Age ^a^	64.53 ± 12.04
Gender ^a^	
Male	55 (49.1%)
Female	57 (50.9%)
Type of therapy ^a^	
Chemotherapy	68 (60.7%)
Immunotherapy	23 (20.5%)
Chemotherapy and Immunotherapy	16 (14.3%)
Targeted Therapy	3 (2.7%)
Type of Cancer ^a^	
Solid tumors	111 (99.1%)
Hematological cancer	1 (0.9%)
Living Situation ^a^	
Home	112 (100%)
Time taken to complete FFQ ^b^	9.96 ± 2.60

^a^*n* = 112. ^b^ *n* = 91.

**Table 2:** Summary of results from Bland–Altman analysis demonstrating agreement between Food Frequency Questionnaire (FFQ) and diet history for 14 different micronutrients. Bias and standard deviation (SD) with 95% confidence intervals (95% CI), lower and upper limits of agreement (LOA) with respective 95% CI, and clinically acceptable bias and LOA ranges are presented.

Nutrient	Bias ± SD (95% CI)	Lower LOA (95% CI)	Upper LOA (95% CI)	Clinically Acceptable Bias (±) ^a^	Clinically Acceptable LOA (±) ^b^
Copper (mg)	0.00 * ± 0.49 (−0.10, 0.09)	−0.97 ^c^ (−1.13, −0.81)	0.96 ^c^ (0.80, 1.12)	0.55	1.10
Iron (mg)	−0.73 * ± 5.72 (−1.80, 0.34)	−11.93 (−13.79, −10.08)	10.47 (8.62, 12.33)	4.08	8.16
Zinc (mg)	−1.13 * ± 3.01 (−1.69, −0.56)	−7.03 (−8.01, −6.05)	4.78 (3.80, 5.75)	2.10	4.20
Retinol Equivalents (µg)	361.52 * ± 1911.10 (3.69, 719.36)	−3384.16 ^c^ (−4003.95, −2764.38)	4107.21 ^c^ (3487.42, 4727.00)	2395.98	4791.95
Vitamin C (mg)	79.23 ± 119.99 (56.76, 101.69)	−155.95 (−194.87, −117.04)	314.40 (275.49,353.32)	48.77	97.54
Cholecalciferol (D3) (µg)	0.45 * ± 1.49 (0.17, 0.73)	−2.46 ^c^ (−2.94, −1.98)	3.37 ^c^ (2.88, 3.85)	5.39	10.78
Vitamin E (mg)	1.45 * ± 5.48 (0.43, 2.48)	−9.29 ^c^ (−11.07, −7.51)	12.19 (10.42, 13.97)	4.77	9.54
Alpha Linolenic Acid (g)	0.07 * ± 0.85 (−0.09, 0.22)	−1.59 (−1.87, −1.32)	1.72 (1.45, 2.00)	0.57	1.14
Total LC *n*-3 FA (mg)	−8.59 * ± 460.46 (−94.81, 77.62)	−911.09 ^c^ (−1060.42, −761.75)	893.90 ^c^ (744.57, 1043.23)	1085.91	2171.82
Arginine (mg)	148.95 * ± 766.31 (5.47, 292.44)	−1352.99 ^c^ (−1601.52, −1104.47)	1650.90 ^c^ (1402.38, 1899.42)	1372.01	2744.03
Glutamic Acid (mg)	249.60 * ± 1892.85 (−104.82, 604.02)	−3460.32 ^c^ (−4074.19, −2846.45)	3959.52 ^c^ (3345.65, 4573.39)	2050.85	4101.70
Isoleucine (mg)	31.68 * ± 569.12 (−74.88,138.25)	−1083.78 ^c^ (−1268.35, −899.20)	1147.14 ^c^ (962.57, 1331.71)	994.26	1988.52
Leucine (mg)	43.45 * ± 878.65 (−121.07, 207.97)	−1678.67 ^c^ (−1963.63, −1393.72)	1765.57 ^c^ (1480.62, 2050.53)	1448.07	2896.13
Valine (mg)	74.67 * ± 680.50 (−52.75, 202.08)	−1259.09 ^c^ (−1479.78, −1038.39)	1408.42 ^c^ (1187.73, 1629.11)	1151.57	2303.13

^a^ Clinically acceptable bias is based on foods containing high amounts of respective nutrients per serve. ^b^ Clinically acceptable LOA is based on foods containing high amounts of respective nutrients per 2 serves. * Bias within clinically acceptable range and 95% CI. ^c^ LOA within clinically acceptable range and 95% CI. *N* = 112.

**Table 3:** Summary of results from Passing–Bablok regression test presenting intercept and slope with respective 95% confidence intervals (95% CI) and cumulative sum linearity (cusum linearity) test with respective *p*-values.

Nutrient	Intercept (95% CI)	Slope (95% CI)	H Value	*p*-Value
Copper (mg)	0.20 (0.04, 0.36)	0.80 (0.64,0.98)	0.93	>0.20 ^c^
Iron (mg)	1.20 ^a^ (−0.52, 2.30)	0.87 ^b^ (0.73, 1.04)	1.04	>0.05 ^c^
Zinc (mg)	1.78 (0.38, 3.07)	0.68 (0.54, 0.83)	0.53	>0.20 ^c^
Retinol Equivalents (µg)	245.31 ^a^ (−89.92, 548.05)	1.21 ^b^ (0.86, 1.72)	0.79	>0.20 ^c^
Vitamin C (mg)	5.05 ^a^ (−53.20, 48.12)	1.75 (1.25, 2.52)	1.46	<0.05
Cholecalciferol (D3) (µg)	0.77 (0.52, 0.97)	0.79 ^b^ (0.63, 1.04)	1.19	>0.10 ^c^
Vitamin E (mg)	0.75 ^a^ (−1.27, 2.76)	1.01 ^b^ (0.78, 1.38)	0.79	>0.20 ^c^
Alpha Linolenic Acid (g)	0.11 ^a^ (−0.04, 0.26)	0.90 ^b^ (0.72, 1.15)	1.19	>0.10 ^c^
Total LC *n*-3 FA (mg)	46.5 ^a^ (−4.49, 84.74)	0.92 ^b^ (0.73, 1.17)	1.19	>0.10 ^c^
Arginine (mg)	311.54 (110.37, 486.65)	0.90 ^b^ (0.73, 1.11)	1.06	>0.20 ^c^
Glutamic Acid (mg)	680.15 (46.56, 1129.52)	0.86 ^b^ (0.72, 1.06)	1.32	>0.05 ^c^
Isoleucine (mg)	204.61 (104.47, 319.41)	0.80 (0.67,0.97)	1.46	<0.05
Leucine (mg)	347.46 (76.69, 492.19)	0.78 (0.65, 0.97)	1.59	<0.02
Valine (mg)	226.68 (67.68, 379.35)	0.85 ^b^ (0.71, 1.03)	1.46	<0.05

^a^ 95% CI for intercept includes the value 0 indicating no constant difference. ^b^ 95% CI for slope includes the value 1 indicating no proportional difference. ^c^ cusum test *p* value > 0.05 indicating no significant difference from linearity. *N* = 112.

## Data Availability

The data presented in this study are available on request from the corresponding author. The data are not publicly available to maintain privacy of study participants.

## Appendix A

Diet Questionnaire

This questionnaire asks about the types of food you have eaten in the last 12 months.

This includes all foods and drinks you have eaten for meals, in-between meals, and away from home.

Every question should be answered, even if it is just an estimate.

Over the last 12 months how much of the following foods did you eat on average each day?

1. Bread (1 slice bread = ½ bread roll)

☐ None ☐ ½ slice ☐ 1 slice ☐ 2 slices ☐ 3 slices ☐ 4 slices ☐ 5 slices ☐ 6 slices

What type of bread?

☐ Whole grain ☐ Wholemeal ☐ White

2. Milk, including on its own as a beverage, with cereal, and as an ingredient to drinks such as tea or coffee (1 cup = 250 mL)

☐ None ☐ ½ cup ☐ 1 cup ☐ 1½ cup ☐ 2 cups ☐ 2½ cups ☐ 3 cups ☐ 4 cups

What type of milk?

☐ Full fat ☐ Reduced fat ☐ Skim

3. Margarine (Flora, Nuttelex, MeadowLea, Olive Grove) including spread on bread or toast, and on vegetables (1 teaspoon (thin spread) = 5 g; 4 teaspoons = 20 g = 1 tablespoon (thick spread))

☐ None ☐ 1 tsp ☐ 2 tsp ☐ 3 tsp ☐ 1 Tbs ☐ 2 Tbs ☐ 3 Tbs ☐ 4 Tbs

What type of margarine?

☐ Flora or Nuttelex ☐ MeadowLea ☐ Olive Grove

4. Oil in cooking, on vegetables or salad, or on bread or toast (1 teaspoon = 5 g; 4 teaspoons = 20 g = 1 tablespoon) Please estimate the amount that was in your meal alone.

☐ None ☐ 1 tsp ☐ 2 tsp ☐ 3 tsp ☐ 1 Tbs ☐ 2 Tbs ☐ 3 Tbs

What type of oil?

☐ Sunflower oil ☐ Canola or vegetable oil ☐ Olive oil

5. Fruit

5.1 Apple, pear, banana, grapes (1 serve = 1 whole medium apple, pear, banana or 1 cup grapes or 2/3 cup tinned fruit)

☐ None ☐ ¼ serve or 1 serve 2/wk ☐ ½ serve or 1 serve 3–4/wk☐ 1 serve ☐2 serves ☐ 3 serves ☐ 4 serves ☐ 5 serves

5.2. Orange, mandarin, kiwi fruit, berries (1 serve = 1 whole orange or 2 small mandarins/kiwi fruit or 1 cup berries)

☐ None ☐ ¼ serve or 1 serve 2/wk ☐ ½ serve or 1 serve 3–4/wk ☐ 1 serve ☐2 serves ☐ 3 serves ☐ 4 serves ☐ 5 serves

Please answer the following questions for each food type:

(a) Over the last 12 months, on average how often did you eat this food in a week?

(b) Over the last 12 months, on average how much of the following foods did you eat in a single sitting?

If you answer ‘never’ to (a), please skip to the next section.

6. Juice

(a) Times per week

☐ Never ☐ 1/week ☐ 2/week ☐ 3/week ☐ 4/week ☐ 5/week ☐6/week ☐7/week

(b) Portion in one sitting (1 cup = 200 mL)

☐ ¼ cup ☐ ½ cup ☐ 1 cup ☐ 1½ cups ☐ 2 cups ☐ 3 cups ☐ 4 cups

7. Breakfast cereal

(a) Times per week

☐ Never ☐ 1/week ☐ 2/week ☐ 3/week ☐ 4/week ☐ 5/week ☐6/week ☐7/week

What type of cereal?

☐ Oats, porridge, or muesli ☐ Other (rice-, corn-, bran-, wheat-based cereals)

(b) Portion in one sitting (1 serve = 1 cup flaked cereal or 2 Weet-Bix™ or ½ cup oats or muesli)

☐ ½ serve ☐ 1 serve ☐ 2 serves ☐ 3 serves ☐ 4 serves ☐ 5 serves ☐ 6 serves

8. Rice, pasta, or noodles

(a) Times per week

☐ Never ☐ 1/week ☐ 2/week ☐ 3/week ☐ 4/week ☐ 5/week ☐6/week ☐7/week

(b) Portion in one sitting (½ cup = 1 small handful)

☐ ¼ cup ☐ ½ cup ☐ 1 cup ☐ 1½ cups ☐ 2 cups ☐ 2½ cups ☐ 3 cups

9. Potato

(a) Times per week

☐ Never ☐ 1/week ☐ 2/week ☐ 3/week ☐ 4/week ☐ 5/week ☐6/week ☐7/week

(b) Portion in one sitting (1 serve = ½ medium potato or ¼ cup mash)

☐ ½ serve ☐ 1 serve ☐ 2 serves ☐ 3 serves ☐ 4 serves ☐ 5 serves ☐ 6 serves

10. Sweet potato or carrot

(a) Times per week

☐ Never ☐ 1/week ☐ 2/week ☐ 3/week ☐ 4/week ☐ 5/week ☐ 6/week ☐7/week

(b) Portion in one sitting (1 serve = 1 large carrot or ½ medium sweet potato)

☐ ¼ serve ☐ ½ serve ☐ 1 serve ☐ 2 serves ☐ 3 serves ☐ 4 serves ☐ 5 serves

11. Tomato and tomato sauces e.g., pasta sauce

(a) Times per week

☐ Never ☐ 1/week ☐ 2/week ☐ 3/week ☐ 4/week ☐ 5/week ☐6/week ☐7/week

(b) Portion in one sitting (1 serve = 120 g = 1 medium tomato or ½ cup tomato pasta sauce)

☐ ¼ serve ☐ ½ serve ☐ 1 serve ☐ 2 serves ☐ 3 serves ☐ 4 serves ☐ 5 serves

12. Leafy green vegetables e.g., lettuce, spinach, silver beet

(a) Times per week

☐ Never ☐ 1/week ☐ 2/week ☐ 3/week ☐ 4/week ☐ 5/week ☐6/week ☐7/week

(b) Portion in one sitting (1 serve = 1 cup raw or ½ cup cooked)

☐ ¼ serve ☐ ½ serve ☐ 1 serve ☐ 2 serves ☐ 3 serves ☐ 4 serves ☐ 5 serves

13. Green peas—from fresh, frozen, or canned

(a) Times per week

☐ Never ☐ 1/week ☐ 2/week ☐ 3/week ☐ 4/week ☐ 5/week ☐ 6/week ☐ 7/week

(b) Portion in one sitting (½ cup = 1 small handful)

☐ 2 Tbs ☐ ¼ cup ☐ ½ cup ☐ 1 cup ☐ 2 cup

14. Cruciferous vegetables e.g., cauliflower, cabbage, broccoli, Brussel sprouts, Bok choy

(a) Times per week

☐ Never ☐ 1/week ☐ 2/week ☐ 3/week ☐ 4/week ☐ 5/week ☐ 6/week ☐ 7/week

(b) Portion in one sitting (1 serve = 1 cup raw or ½ cup cooked)

☐ ¼ serve ☐ ½ serve ☐ 1 serve ☐ 2 serves ☐ 3 serves ☐ 4 serves ☐ 5 serves

15. Beef, veal, lamb, or pork, including as roast, steak, mince, chops, stew etc.

(a) Times per week

☐ Never ☐ 1/week ☐ 2/week ☐ 3/week ☐ 4/week ☐ 5/week ☐ 6/week ☐ 7/week

(b) Portion in one sitting (1 serve = 65 g = ½ cup mince or 1 chop or 1 slice roast meat or 1 sausage)

☐ ½ serve ☐ 1 serve ☐ 2 serves ☐ 3 serves ☐ 4 serves ☐ 5 serves

16. Chicken or other poultry

(a) Times per week

☐ Never ☐ 1/week ☐ 2/week ☐ 3/week ☐ 4/week ☐ 5/week ☐ 6/week ☐ 7/week

(b) Portion in one sitting (1 serve = 80 g = ½ breast fillet or 1 chicken drumstick or 2/3 chicken thigh)

☐ ½ serve ☐ 1 serve ☐ 2 serves ☐ 3 serves ☐ 4 serves ☐ 5 serves ☐ 6 serves

17. Oily fish e.g., salmon, tuna, sardines, mackerel

(a) Times per week

☐ Never ☐ 1/week ☐ 2/week ☐ 3/week ☐ 4/week ☐ 5/week ☐ 6/week ☐ 7/week

(b) Portion in one sitting (1 serve = 100 g = 1 fillet or 1 small can)

☐ ½ serve ☐ 1 serve ☐ 2 serves ☐ 3 serves ☐ 4 serves ☐ 5 serves ☐ 6 serves

What type of fish?

☐ Salmon ☐ Tuna ☐ Sardines ☐ Mackerel

18. Other white fish e.g., whiting, flathead

(a) Times per week

☐ Never ☐ 1/week ☐ 2/week ☐ 3/week ☐ 4/week ☐ 5/week ☐ 6/week ☐ 7/week

(b) Portion in one sitting (1 serve = 100 g = 1 fillet)

☐ ½ serve ☐ 1 serve ☐ 2 serves ☐ 3 serves ☐ 4 serves ☐ 5 serves ☐ 6 serves

19. Eggs, including egg dishes such as omelette or frittata

(a) Times per week

☐ Never ☐ 1/week ☐ 2/week ☐ 3/week ☐ 4/week ☐ 5/week ☐ 6/week☐ 7/week

(b) Portion in one sitting

☐ 1 egg ☐ 2 eggs ☐ 3 eggs ☐ 4 eggs ☐ 5 eggs ☐ 6 eggs

20. Cheese

(a) Times per week

☐ Never ☐ 1/week ☐ 2/week ☐ 3/week ☐ 4/week ☐ 5/week ☐ 6/week☐ 7/week

(b) Portion in one sitting (1 serve = 40 g or 2 slices hard cheese)

☐ ½ serve ☐ 1 serve ☐ 2 serves ☐ 3 serves ☐ 4 serves ☐ 5 serves ☐ 6 serves

21. Nuts e.g., loose nuts or nuts spread such as peanut butter

(a) Times per week

☐ Never ☐ 1/week ☐ 2/week ☐ 3/week ☐ 4/week ☐ 5/week ☐ 6/week ☐ 7/week

(b) Portion in one sitting (1 serve = 1 handful or 30 g or 2 tablespoons nut spread)

☐ ½ serve ☐ 1 serve ☐ 2 serves ☐ 3 serves ☐ 4 serves ☐ 5 serves ☐ 6 serves

What type of nuts?

☐ Walnuts ☐ Almonds ☐ Peanuts ☐ Cashews ☐ Pistachio ☐ Other

Do you currently take any nutritional supplements?

☐ No

☐ Yes, please specify the type and dose
